# Modeling Pneumonic Plague in Human Precision-Cut Lung Slices Highlights a Role for the Plasminogen Activator Protease in Facilitating Type 3 Secretion

**DOI:** 10.1128/IAI.00175-19

**Published:** 2019-07-23

**Authors:** Srijon K. Banerjee, Samantha D. Huckuntod, Shalynn D. Mills, Richard C. Kurten, Roger D. Pechous

**Affiliations:** a Department of Microbiology and Immunology, University of Arkansas for Medical Sciences, Little Rock, Arkansas, USA; b Department of Physiology and Biophysics, University of Arkansas for Medical Sciences, Little Rock, Arkansas, USA; c Lung Cell Biology Laboratory, Arkansas Children’s Research Institute, Little Rock, Arkansas, USA; Yale University School of Medicine

**Keywords:** Pla, *Yersinia*, *Yersinia pestis*, hPCLS, human precision-cut lung slices, plague, plasminogen activator protease, pneumonic plague, pulmonary infection, type 3 secretion

## Abstract

Pneumonic plague is the deadliest form of disease caused by Yersinia pestis. Key to the progression of infection is the activity of the plasminogen activator protease Pla. Deletion of Pla results in a decreased Y. pestis bacterial burden in the lung and failure to progress into the lethal proinflammatory phase of disease. While a number of putative functions have been attributed to Pla, its precise role in the pathogenesis of pneumonic plague is yet to be defined. Here, we show that Pla facilitates type 3 secretion into primary alveolar macrophages but not into the commonly used THP-1 cell line. We also establish human precision-cut lung slices as a platform for modeling early host/pathogen interactions during pneumonic plague and solidify the role of Pla in promoting optimal type 3 secretion using primary human tissue with relevant host cell heterogeneity. These results position Pla as a key player in the early host/pathogen interactions that define pneumonic plague and showcase the utility of human precision-cut lung slices as a platform to evaluate pulmonary infection by bacterial pathogens.

## Abstract (precis)

Pneumonic plague is the deadliest form of disease caused by Yersinia pestis. Key to the progression of infection is the activity of the plasminogen activator protease Pla. Deletion of Pla results in a decreased Y. pestis bacterial burden in the lung and failure to progress into the lethal proinflammatory phase of disease. While a number of putative functions have been attributed to Pla, its precise role in the pathogenesis of pneumonic plague is yet to be defined.

## INTRODUCTION

Yersinia pestis causes bubonic, septicemic, and pneumonic plague and is one of nature’s deadliest pathogens. Inoculation via a bite from an infected flea results in bubonic plague, the most common form of disease. Inhalation of contaminated droplets containing Y. pestis results in primary pneumonic plague, the most lethal manifestation of Y. pestis infection. Pneumonic plague is fatal in 4 to 7 days unless antibiotics are administered within 24 h after the onset of symptoms (1, 2). The lethality, ability to be transmitted via the aerosol route, and pandemic potential of Y. pestis have resulted in its designation as a tier 1 select agent and compound fears of its intentional release (1). The threat of Y. pestis in the modern era was evident in the 2017 Madagascar outbreak, which saw over 2,000 confirmed cases of plague, 1,791 of which were pneumonic plague (3–5).

Key to the progression of pneumonic plague is an early preinflammatory disease phase, during which the bacteria survive and proliferate in the lungs in the absence of symptoms or signs of inflammation (6, 7). Though the precise mechanism for establishing a preinflammatory phase remains unclear, secretion of the *Yersinia* outer proteins (Yops) into target host cells using a type 3 secretion system (T3SS) is required (6, 8). The preinflammatory phase lasts roughly 2 to 4 days, after which infection abruptly progresses into a proinflammatory state with massive innate immune cell infiltration into the airways and the onset of a proinflammatory cytokine storm (6, 9). Uncontrolled inflammation in the lungs ultimately compromises pulmonary function and results in death.

Y. pestis virulence is largely attributed to the Ysc T3SS, encoded on the plasmid pCD1, and a handful of key virulence factors, including the plasminogen activator (Pla) protease (10). Pla is an omptin family aspartic protease that cleaves plasminogen into plasmin, which promotes the degradation of fibrin clots (11, 12). Though Pla is required for the progression of both bubonic and pneumonic plague, its function appears to differ between the two disease types. Pla facilitates the dissemination of Y. pestis from the initial flea bite into deeper tissue during bubonic plague but is not essential for growth at the site of inoculation (13, 14). In contrast, during pneumonic plague, deletion of Pla significantly impairs bacterial growth in the lung but does not inhibit dissemination to other tissues (15). Deletion of Pla results in attenuation of Y. pestis, which can be detected as early as 24 h postinfection (hpi), suggesting that Pla plays a role very early after pulmonary infection (15). The function of Pla that promotes bacterial survival during early events in the lung that establish pneumonic plague is not known.

Modeling the initial host/pathogen interactions that occur in the alveolar spaces is difficult using standard infection platforms. Human precision-cut lung slices (hPCLS) are a recently developed tool that have shown utility for modeling the human respiratory tract and serve as a powerful platform to complement existing *in vitro* and *in vivo* infection models (16, 17). hPCLS are slices of living tissue obtained from donor lungs that serve as a three-dimensional organotypic model. hPCLS can be maintained under standard tissue culture conditions (18) and are responsive to pharmacological and biological treatment (19). While hPCLS have primarily been used to evaluate drug toxicity and model airway constriction (18–20), recent work evaluating infection of hPCLS with the bacterium Coxiella burnetii highlights their utility for examining host/pathogen interactions during pulmonary infection (16). In this study, we establish hPCLS as an infection platform to evaluate primary pneumonic plague. We used hPCLS in tandem with primary human alveolar macrophages (hAMs) to examine the role of Pla in early interactions of Y. pestis with alveolar macrophages. We show that Pla facilitates optimal type 3 secretion (T3S), primarily into alveolar macrophages, and that its absence results in increased proinflammatory cytokine secretion. We confirmed this finding using Y. pestis lacking Pla in a mouse intranasal infection model. This work uses a novel and highly relevant human infection platform to further define the role of a key *Yersinia* virulence factor that is essential to the progression of primary pneumonic plague.

## RESULTS

### Pla contributes to adherence to and T3S into primary human alveolar macrophages.

**FIG 1:**
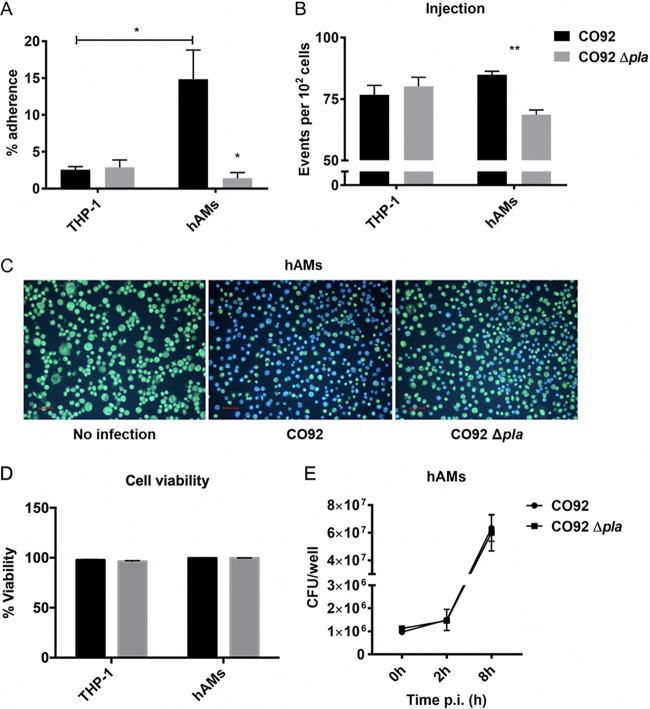
Adherence and T3S of Y. pestis wild-type and Δ*pla* strains *in vitro*. (A) THP-1 cells differentiated with PMA and hAMs were seeded at 10^5^/ml and infected with bacteria at an MOI of 20 for 1 h. Cells were washed and plated on BHI for CFU enumeration. Control wells were not washed, and the total bacteria in the well were plated. Plots represent the comparison between washed and control wells. (B) Yop translocation by CO92 and CO92 Δ*pla* as measured by flow cytometry. THP-1 cells and hAMs were seeded at 5 × 10^5^/ml and infected with the wild-type and Δ*pla* strains harboring a YopE-Bla reporter protein at an MOI of 20 for 1 h. Cells were treated with CCF2-AM and processed for flow cytometry analysis. (C) Epifluorescence microscopy of hAMs to detect the difference in Yop translocation between the wild-type and Δ*pla* strains of Y. pestis. hAMs were seeded at 5 × 10^5^ cells/ml and infected with the wild-type and Δ*pla* strains at an MOI of 20 for 1 h. Cells were treated with CCF2-AM and processed for microscopy. Blue cells are positive for Yop translocation. Bars, 50 μm. (D) The viability of cells from the experiment whose results are presented in panel C was measured as the percentage of cells that were CCF2-AM positive out of all cells analyzed. (E) Bacterial survival was measured by analysis of the number of CFU after infection of 10^5^ cells/ml at an MOI of 10 for 1 h. For all experiments, *n* was equal to 3 technical replicates (wells). The plots are representative of those from 3 independent experiments. Error bars represent SD. p.i., postinfection. Significance was calculated with Welch’s *t* test; *, *P* < 0.05; **, *P* < 0.01.

Pla has been shown to bind components of the extracellular matrix (ECM) *in vitro* via a mechanism that is distinct from its enzymatic activity (21–23). Also, addition of Pla to Y. pestis lacking all five known adhesins partially restores adherence and Yop delivery to macrophages derived from the human monocytic cell line THP-1 and human epithelial type 2 (HEp-2) cells, suggesting that Pla may contribute to adherence and Yop translocation *in vitro* (24, 25). While complementation with Pla facilitates T3S in the absence of known adhesins, deletion of Pla alone has no effect on adherence or Yop delivery (25). Y. pestis initially targets alveolar macrophages for T3S in the lung during pulmonary infection (26). The finding that deletion of Pla impairs Y. pestis growth in the lung at 24 hpi suggests that Pla contributes to early host/pathogen interactions during pneumonic plague, specifically, the targeting of alveolar macrophages for T3S. As deletion of Pla does not alter adherence to and/or secretion into THP-1 cells, we sought to examine whether Pla facilitates binding to a more physiologically relevant cell type. To this end, we infected primary human alveolar macrophages (hAMs) derived from donor bronchoalveolar lavage fluid (BALF) and evaluated Y. pestis adherence. Similar to previously reported data, deletion of Pla had no effect on adherence to THP-1 cells using a standard adherence assay (Fig. 1A) (25). Surprisingly, wild-type Y. pestis exhibited significantly increased adherence to primary hAMs compared to THP-1 cells, and deletion of Pla resulted in a significant decrease in adherence (Fig. 1A). These results suggest that Pla plays a role in adherence to alveolar macrophages that may not be detected using immortalized cell lines.

To determine if the decreased adherence of Y. pestis to hAMs affects the levels of T3S, we investigated the ability of Y. pestis Δ*pla* to deliver Yops into target cells using Y. pestis YopE-Bla as a reporter. This strain expresses wild-type YopE and a hybrid protein consisting of the first 100 amino acids of YopE fused to a truncated TEM β-lactamase (Bla) and has been used extensively to identify and isolate cells targeted for Yop translocation during infection (26–28). Briefly, upon incubation of cells with the fluorescent substrate coumarin cephalosporin fluorescein acetoxymethyl ester (CCF2-AM), the presence of TEM β-lactamase in cells that have been targeted for YopE-Bla translocation results in blue fluorescence (450 nm) due to β-lactamase cleavage of CCF2-AM, while cells left untargeted fluoresce green (530 nm). We incubated macrophages derived from THP-1 cells and hAMs isolated from donor BALF for 1 h with the Y. pestis wild-type or Δ*pla* strain containing the YopE-Bla reporter to evaluate the delivery of YopE. We observed no difference in the levels of YopE translocation between the wild-type and Δ*pla* strains during infection of THP-1 cells (Fig. 1B). In contrast, infection of hAMs resulted in a significant decrease in T3S by the Δ*pla* strain compared to wild-type Y. pestis (Fig. 1B). Epifluorescence microscopy of hAMs confirmed attenuated Yop delivery by the Δ*pla* strain (Fig. 1C). The attenuated delivery of Yops was not due to altered cell viability of hAMs (Fig. 1D) or attenuated growth of the Δ*pla* strain (Fig. 1E). Interestingly, while infection of hAMs resulted in a roughly 12% increase in adherence compared to that to THP-1 cells, the increase in T3S was not statistically significant (77% for THP-1 cells and 84% for hAMs). A likely explanation for this is that multiple bacteria can bind to and target a single cell, thus slightly skewing the correlation of adherence to injection. Nevertheless, the increased adherence to hAMs and the decreased delivery of Yops in the absence of Pla were clearly observed. In summary, deletion of Pla results in decreased adherence to and T3S into primary alveolar macrophages but not macrophages derived from THP-1 cells. These results suggest that Pla contributes to Y. pestis targeting of alveolar macrophages for T3S during pneumonic plague. In addition, Y. pestis exhibited significantly greater adherence to hAMs than to macrophages derived from THP-1 cells.

### Establishing hPCLS to evaluate the role of Pla in early host/pathogen interactions during pneumonic plague.

**FIG 2:**
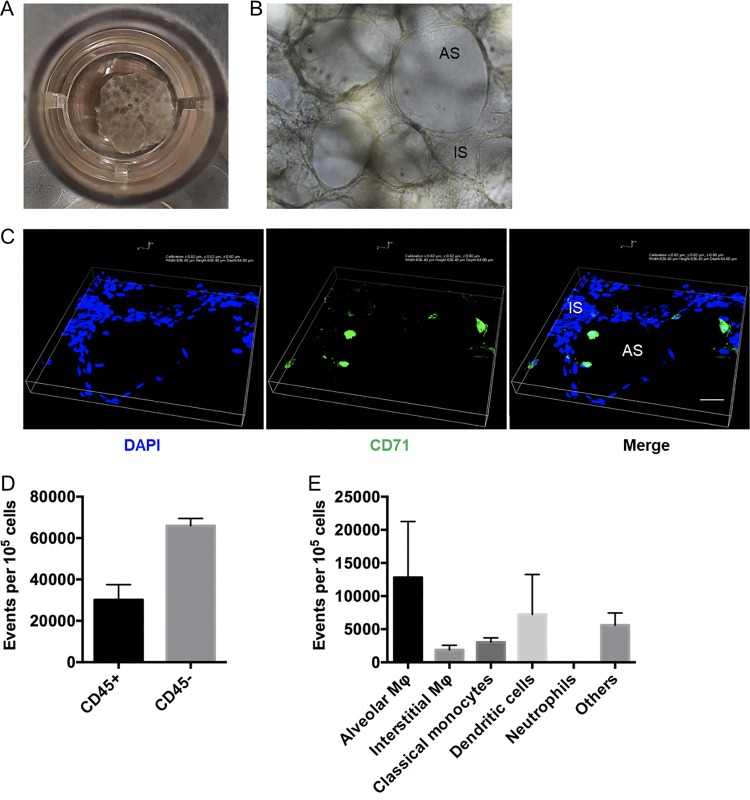
Establishing human precision-cut lung slices as a platform to evaluate Y. pestis Δ*pla*. (A) A single human precision-cut lung slice (hPCLS) with culture medium in one well of a 48-well plate. (B) Bright-field image of hPCLS at ×200 magnification. AS, alveolar space; IS, interstitial space. (C) Immunostaining of lung slices. hPCLS were washed with PBS, fixed, and immunostained with anti-human CD71 conjugated to Alexa Fluor 488 to detect alveolar macrophages. DAPI (4′,6-diamidino-2-phenylindole) was used to stain all cells. Bar, 100 μm. (D, E) Analysis of the total cell types present in hPCLS. (D) hPCLS were digested with collagenase, incubated with fluorescently labeled antibodies to CD45, and analyzed by flow cytometry to determine the ratio of CD45^+^ lymphocyte/leukocyte populations to CD45^−^ epithelial/endothelial populations. (E) CD45^+^ cell types present in hPCLS. The plot represents the population of cells in every 10^5^ CD45^+^ cells that were alveolar macrophages (CD206^+^ CD71^+^), interstitial macrophages (CD206^+^ CD71^−^), classical monocytes (CD206^−^ CD71^−^ CD14^+^), dendritic cells (CD206^−^ CD14^−^ CD11c^+^), neutrophils (CD206^−^ CD24^+^ CD16^+^), or others (other CD45^+^ cells). For all experiments, *n* was equal to 3 technical replicates (wells). The plots are representative of those from 3 independent experiments.

**FIG 3:**
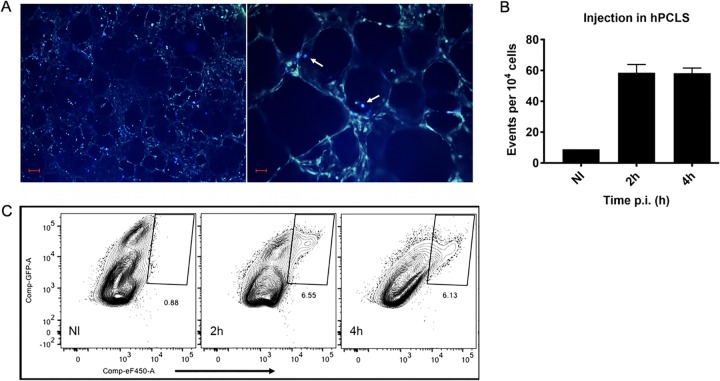
Yop translocation in hPCLS. (A) Epifluorescence microscopy of hPCLS infected with *pgm*-negative CO92 containing the YopE-Bla reporter as a proof of concept. hPCLS was infected with 10^7^ bacteria, washed, and treated with CCF2-AM for microscopy. The two images represent different magnifications: ×40 (left) and ×100 (right). White arrows indicate blue cells that have been targeted for Yop delivery. Bars, 250 μm (left) and 100 μm (right). (B) Time course analyses of Yop translocation in hPCLS infected with 10^7^ CFU of *pgm*-negative CO92 YopE-Bla. hPCLS were washed, digested, and treated with CCF2-AM for flow cytometry. NI, noninfected hPCLS or background. The plot represents the number of blue cells (Yop targeted) in every 10^4^ total cells of the hPCLS. (C) Flow cytometry plots from the experiment whose results are presented in panel B showing the gating for blue cells (Yop targeted). For all experiments, *n* was equal to 3 technical replicates (wells). The plots are representative of those from 3 independent experiments. Error bars represent SD. GFP, green fluorescent protein. Significance was calculated with Welch’s *t* test.

While the use of immortalized cell lines has been critical to our understanding of *Yersinia* pathogenesis, standard tissue culture models lack cell heterogeneity and pulmonary microanatomy and may misrepresent the physiology of the representative cell type. The finding that Y. pestis shows increased adherence to primary hAMs but not macrophages derived from THP-1 cells and the finding that deletion of Pla results in an adherence and T3S defect during infection of hAMs but not THP-1 cells suggest that primary cells and tissue must be used for further analysis. For a more detailed assessment of early host/pathogen interactions during pneumonic plague, we implemented a human precision-cut lung slice (hPCLS) platform to evaluate infection. hPCLS are ∼500- to 700-μm-thick slices of tissue obtained from donor lungs that can be seeded into the wells of tissue culture plates and maintained in the laboratory for several weeks (Fig. 2A). As evidenced by microscopic evaluation, hPCLS maintain the pulmonary microarchitecture, and it is possible to observe both intact alveolar and interstitial spaces (Fig. 2B and C). hPCLS also harbor the full repertoire of host cell types, allowing for quantitation and identification of populations including (but not limited to) macrophages, monocytes, dendritic cells, and CD45^−^ endothelial/epithelial cells (Fig. 2C to E). hPCLS have been used to study the immune response to lipopolysaccharide (LPS) (18), chemical allergens, immunomodulators (19), and pulmonary fibrosis (20) but have only recently been used to examine pulmonary infection (16). To confirm that hPCLS are amenable to analysis with the YopE-Bla/CCF2-AM reporter system, hPCLS in wells of a 48-well plate were infected with a *pgm*-negative Y. pestis CO92 strain containing the YopE-Bla reporter to evaluate Yop delivery. Figure 3A shows representative epifluorescence images (×40 and ×100 magnifications) of an infected hPCLS, with cells that have been targeted for YopE-Bla translocation exhibiting blue fluorescence. Flow cytometry analysis demonstrated T3S of hPCLS at 2 and 4 hpi (Fig. 3B and C), highlighting the utility of the hPCLS as a platform for evaluating Y. pestis infection and T3S.

### Deletion of Pla alters Y. pestis T3S in hPCLS.

**FIG 4:**
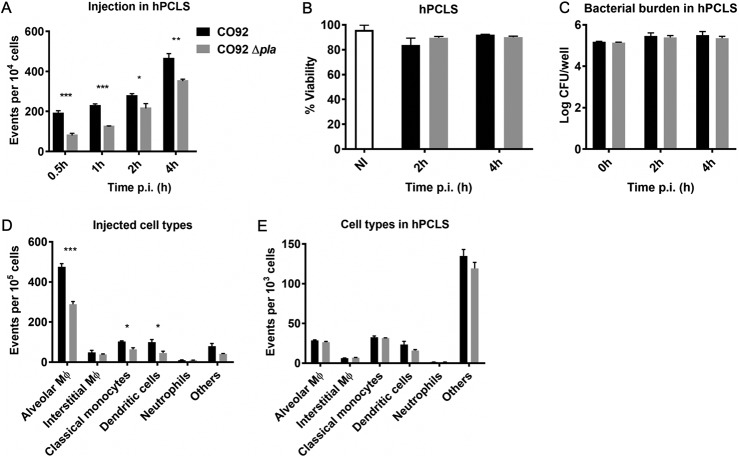
T3S of Y. pestis wild-type and Δ*pla* strains in hPCLS. (A) Flow cytometry analyses of Yop translocation levels in hPCLS infected with CO92 and CO92 Δ*pla* containing the YopE-Bla reporter. hPCLS were infected with 10^7^ bacteria for 30 min or 1 h, washed, and either overlaid with medium for longer times or digested, stained with CCF2-AM, and fixed for analysis. The plot represents the number of blue cells (Yop targeted) in every 10^4^ cells of the hPCLS. (B) Viability of hPCLS cells after infection with Y. pestis. hPCLS were infected with 10^7^ CFU of CO92 and CO92 Δ*pla* for 1 h, washed, and overlaid with medium. At the indicated time points, hPCLS were digested and stained with CCF2-AM to measure viability (green fluorescence, CCF2-AM positive) via flow cytometry. (C) Bacterial burden in hPCLS. Slices were infected with 10^5^ CFU of CO92 and CO92 Δ*pla*. At the indicated time points, hPCLS were digested and the contents of the well were serially diluted to enumerate the CFU. (D) Cell types targeted for Yop delivery by Y. pestis lacking Pla. hCPLS were infected with 10^7^ CFU of the Y. pestis YopE-Bla wild-type and Δ*pla* strains for 1 h, digested, and stained with fluorescently labeled antibodies. The plot represents the population of blue cells (Yop targeted) in every 10^5^ of CD45^+^ cells that were alveolar macrophages (CD206^+^ CD71^+^), interstitial macrophages (CD206^+^ CD71^−^), classical monocytes (CD206^−^ CD71^−^ CD14^+^), dendritic cells (CD206^−^ CD14^−^ CD11c^+^), neutrophils (CD206^−^ CD24^+^ CD16^+^), or others (other CD45^+^ cells). (E) Analysis of the total cell types present in infected hPCLS. hCPLS were infected with 10^7^ CFU of the Y. pestis YopE-Bla wild-type and Δ*pla* strains treated as described in the legend to panel D. The plot represents the total population of alveolar macrophages, interstitial macrophages, classical monocytes, dendritic cells, neutrophils, and other CD45^+^ cells in the infected lung slices. For all experiments, *n* was equal to 3 technical replicates (wells). The plots are representative of those from 3 independent experiments. Error bars represent SD. Significance was calculated with Welch’s *t* test. *, *P* < 0.05; **, *P* < 0.01; ***, *P* < 0.001.

We next sought to evaluate the role of Pla in T3S using hPCLS, which allows for interrogation of a number of relevant host cell types simultaneously. Infection of hPCLS with the Δ*pla* strain resulted in significantly reduced Yop translocation early after infection and continuing to 4 hpi (Fig. 4A). Importantly, the reduction in the levels of T3S was not due to changes in hPCLS viability or the bacterial burden resulting from deletion of Pla (Fig. 4B and C). Using a panel of fluorescently labeled antibodies and flow cytometry, we sought to determine whether the defect in T3S in the absence of Pla was due to decreased T3S in general or reduced targeting of a specific cell type. Y. pestis preferentially targets professional phagocytes for T3S, specifically, alveolar macrophages, in a murine infection model (26, 27). Staining of CD45^+^ cells from infected hPCLS with a panel of fluorescently labeled antibodies (29) revealed that translocation was significantly reduced in alveolar macrophages for the Δ*pla* strain compared to that for the wild-type Y. pestis strain (Fig. 4D). While dendritic cells and monocytes also appear to have received reduced levels of Yops from the Δ*pla* strain, the overall numbers of those cells targeted for translocation were significantly lower than the overall numbers of alveolar macrophages targeted (Fig. 4D). Therefore, we reasoned that the overall reduction of T3S by Y. pestis Δ*pla* in hPCLS could be largely attributed to reduced T3S into alveolar macrophages. There was no significant difference in T3S for any other CD45^+^ cells examined. Total alveolar macrophage numbers were unchanged in hPCLS infected with wild-type or mutant strains of Y. pestis, indicating that decreased Yop translocation into alveolar macrophages was not due to a change in the host cell repertoire in response to infection with the Δ*pla* strain (Fig. 4E). These data indicate that Pla facilitates optimal T3S in human tissue. As they are the primary target population, decreased translocation into alveolar macrophages is primarily responsible for the overall decrease in T3S.

**FIG 5:**
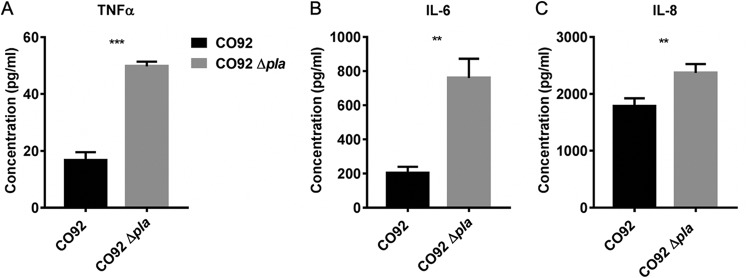
Infection of hPCLS with Y. pestis Δ*pla* alters proinflammatory cytokine secretion. (A to C) Cytokine secretion in hPCLS infected with Y. pestis. hPCLS were infected with 10^7^ CFU of CO92 and CO92 Δ*pla* for 1 h, washed, and overlaid with medium. At the indicated time points, the medium was collected and filtered, and cytokines were measured using bead arrays. TNF-α (A), IL-6 (B), and IL-8 (C) levels after subtraction of the levels for uninfected cells are represented. For all experiments, *n* was equal to 3 technical replicates (wells). The plots are representative of those from 3 independent experiments. Error bars represent SD. Significance was calculated with Welch’s *t* test. **, *P* < 0.01; ***, *P* < 0.001.

Proinflammatory cytokine expression is below the limit of detection during the preinflammatory phase in mouse lungs during both the presence and the absence of Pla (15). Due to the attenuation of Y. pestis lacking Pla in the lung, we predicted that reduced targeting of alveolar macrophages would alter host inflammatory responses to infection that could be detected in hPCLS. The use of hPCLS allows for the direct measurement of bacterial/host cell interactions and abrogates the difficulty of detecting early host responses in whole-tissue or whole-animal analysis during *in vivo* infection. To evaluate how deletion of Pla affects the downstream effects of early host/pathogen interactions in the human lung, hPCLS were infected with the Y. pestis wild-type or Δ*pla* strain and the levels of proinflammatory cytokines were measured in culture supernatants using a cytokine bead array kit. Infection of hPCLS resulted in increased levels of tumor necrosis factor alpha (TNF-α), interleukin-6 (IL-6), and IL-8 in response to the Δ*pla* strain compared to those in response to wild-type Y. pestis at 2 hpi (Fig. 5A to C). These results suggest that Pla contributes to the early inhibition of proinflammatory cytokine expression resulting from host/pathogen interactions that occur in pulmonary tissue. These data also demonstrate the utility of hPCLS in measuring the immediate effects of early host/pathogen interactions in the pulmonary compartment that may be difficult to detect *in vivo*.

### Deletion of Pla results in decreased Y. pestis T3S into alveolar macrophages *in vivo* during pneumonic plague.

**FIG 6:**
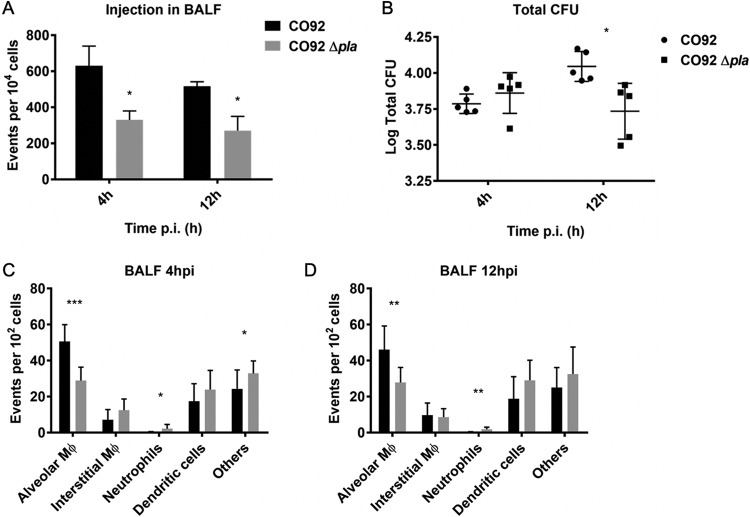
Yop translocation in murine lungs in the presence and absence of Pla. (A) Yop translocation levels in the BALF of C57BL/6 mice infected with 5 × 10^4^ CFU of CO92 and CO92 Δ*pla* containing YopE-Bla for 4 and 12 h. BALF was treated with CCF2-AM and subjected to flow cytometry analysis. The plot represents the number of blue cells (Yop targeted) in every 10^4^ cells of the BALF. (B) Number of bacterial CFU obtained in the lungs of infected C57BL/6 mice after BALF was collected. The plot represents the total combined number of CFU in the BALF and the lavaged lung at both 4 and 12 hpi. (C, D) BALF collected at 4 hpi (C) and 12 hpi (D) in the experiment whose results are presented in panel A was immunostained, stained with CCF2-AM, fixed, and analyzed by flow cytometry to identify the proportion of blue cells that were alveolar macrophages (F4/80^+^ CD11c^high^ CD11b^mid/low^), interstitial macrophages (CD45^+^ CD3^−^ F4/80^+^ CD11c^low^ CD11b^high^), neutrophils (CD45^+^ CD3^−^ F4/80^−^ Ly-6G^+^ CD11b^high^), dendritic cells (CD45^+^ CD3^−^ F4/80^−^ CD11c^high^ CD11b^high/low^), or others (other blue cells). For all experiments, *n* was equal to 5 mice. The plots are representative of those from 3 independent experiments. Error bars represent SD. Significance was calculated with Welch’s *t* test. *, *P* < 0.05; **, *P* < 0.01; ***, *P* < 0.001.

We next sought to confirm whether the finding that the Δ*pla* mutant exhibits reduced Yop translocation during infection of hPCLS and hAMs translates to an *in vivo* infection model of pneumonic plague. To this end, we evaluated Yop translocation in the bronchoalveolar lavage fluid (BALF) of mice infected with wild-type and Δ*pla* strains of Y. pestis expressing YopE-Bla at 4 and 12 hpi. Infection with Y. pestis lacking Pla resulted in a significant defect in Yop translocation compared to that seen with wild-type infection (Fig. 6A). The bacterial numbers of both strains in the lungs were similar at 4 hpi (Fig. 6B), confirming that the difference in translocation was not due to a reduced bacterial burden for Y. pestis Δ*pla*. We next measured T3S in various immune cell populations found in the BALF early after infection. Similar to the findings for hPCLS, Yop translocation was significantly reduced in alveolar macrophages in the absence of Pla early during infection at 4 hpi (Fig. 6C) and at 12 hpi (Fig. 6D). While interstitial macrophages (CD11b^high^ CD11c^low^ macrophages that have recently migrated into the alveolus), neutrophils, dendritic cells, and other cell types (not identified by the antibody panel used in the current study) are targeted for Yop delivery, the reduction in total injection levels is solely due to the decreased delivery of Yops to alveolar macrophages in the absence of Pla. These data indicate that Pla facilitates Y. pestis T3S during the early stages of pneumonic plague in an intranasal murine infection model. The deletion of Pla most dramatically affected targeting of alveolar macrophages, the primary host cell target early after infection.

## DISCUSSION

In the work described here, we demonstrate a clear role for Pla in facilitating targeting of host cells, primarily alveolar macrophages, for T3S in primary human tissue. Intriguingly, Y. pestis is much more adherent to alveolar macrophages than to cells of the commonly used cell line THP-1, and deletion of *pla* results in a significant decrease in adherence to and T3S into hAMs and hPCLS. The decreased T3S of hPCLS in the absence of Pla resulted in increased proinflammatory cytokine production, suggesting that Pla likely contributes to maintenance of an early preinflammatory phase of disease. The decrease in Yop translocation in the absence of Pla translates to an *in vivo* murine infection model, confirming the key role of Pla in the early events that establish infection. This is the first demonstration of Pla facilitating T3S *in vivo* and the first time that deletion of *pla* was shown to result in an adherence and secretion defect in macrophages. In summary, we implicate Pla as a key player in the early host/pathogen interactions that define pneumonic plague.

This work also establishes the use of hPCLS to evaluate the pathogenesis of pneumonic plague. We demonstrated the utility of this novel infection platform by evaluating the role of a key Pla in the earliest host/pathogen interactions that occur in the alveolar spaces. hPCLS have been used to examine immune responses to chemical allergens, LPS, and other immunomodulators (19, 30, 31). Pioneering work by Graham et al. utilized hPCLS to examine the pathogenesis of Coxiella burnetii, the causative agent of Q fever, effectively demonstrating their power as a platform to evaluate key events during pulmonary infection (16). Standard tissue culture models lack appropriate cell heterogeneity and pulmonary microanatomy and may misrepresent the physiology of the representative cell type. This is evidenced by the decreased targeting of hAMs in the absence of Pla, a phenotype that was not apparent during infection of THP-1 cells. During murine infection, it is often difficult to isolate and examine the relatively small number of bacteria and host cells necessary to evaluate early host/pathogen interactions in the lung. hPCLS serve as a physiologically relevant platform for detailed analysis of host/pathogen interactions during pulmonary infection. Further, use of primary human tissue provides insight into differences between human infection and standard animal infection models that may be crucial to our understanding of human disease. Though animal models are essential to understanding infection with Y. pestis, differences impacting pathogenesis in mice compared to that in humans have been revealed (32–34). Along these lines, the applicability of many animal models to human disease has been of recent concern to the NIH, as countless findings have not translated during human clinical trials (35–40). The use of primary human cells and tissue may abrogate these concerns.

Deletion of Pla alters bacterial survival in the lungs but does not prevent dissemination to other tissues (15). It was recently shown that acquisition of Pla is sufficient for ancestral strains of Y. pestis to replicate to high levels in the pulmonary compartment, highlighting its importance in the ability of Y. pestis to cause pneumonic plague (41). A number of functions have been attributed to Pla, including activation of host plasminogen into plasmin and cleavage of a number of substrates, including complement component C3, Fas ligand, peroxiredoxin 6, and α2-antiplasmin (42, 43). To date, Fas ligand is the only substrate for Pla that has been confirmed *in vivo* to potentially contribute to primary pneumonic plague (44). While cleavage of the Fas ligand is thought to modulate host immune responses by inhibiting apoptosis during the later proinflammatory stage of infection (44), the finding that Y. pestis lacking Pla exhibits significantly attenuated growth as early as 24 hpi led us to investigate the role of Pla during the earliest events that define pneumonic plague. Infection of primary hAMs and hPCLS revealed that Y. pestis lacking Pla exhibits a significantly reduced T3S that correlates with decreased adherence. Previous work suggests that Pla binds components of the extracellular matrix, and expression of Pla in Escherichia coli mediates invasion of human endothelial cells (21, 22, 32). The function of Pla that is responsible for adherence to mammalian cells is independent of its proteolytic activity and is capable of facilitating T3S (25, 45). The precise mechanism by which Pla facilitates adherence and T3S remains unknown but clearly involves host factors that are not associated with the THP-1 cells commonly used to model bacterial/macrophage interactions. It was previously suggested that Pla may bind DEC-205 (CD205) to promote invasion of alveolar macrophages (46). Current work is geared toward determining if Pla itself acts as an adhesin, whether its enzymatic activity exposes or processes a key adhesin, or whether Pla is involved in remodeling the cell membrane, allowing for exposure of a specific adhesin.

Yop translocation dramatically alters the ability to mount an effective innate immune defense during infection. For example, YopH suppresses TNF-α and IL-1β in mouse lungs (47), and YopJ inhibits NF-κB signaling (48) and IL-8 secretion (49). The finding that infection of hPCLS with Y. pestis lacking Pla results in increased secretion of IL-8, IL-6, and TNF-α suggests that Pla may play an early immunosuppressive role in the lung by facilitating efficient T3S of alveolar macrophages. To our knowledge, this is the first study establishing the role of Pla as a suppressor of proinflammatory cytokine expression.

We also sought to determine whether the role of Pla in facilitating T3S of alveolar macrophages translates to our standard intranasal mouse infection model. Deletion of Pla resulted in a significant decrease in total Yop translocation in BALF early after infection. Importantly, during the earliest time points after infection, bacterial burdens were equivalent for Y. pestis wild-type and Δ*pla* strains, confirming that the T3S defect is a result of the function of Pla and not a reflection of bacterial survival. Flow cytometry analysis confirmed that, similar to infection in hPCLS, Y. pestis Δ*pla* exhibits an early defect in the targeting of alveolar macrophages for T3S. This is the first time that the absence of Pla has been shown to diminish T3S and the first time that Pla has been shown to contribute to Y. pestis T3S *in vivo* during infection. In summary, this work identifies a mechanism for a key Y. pestis virulence factor that aids in establishing disease in the lung. In addition, we demonstrate the utility of hPCLS for modeling pneumonic plague and highlight the power of primary human cells and tissues as a platform to evaluate host/pathogen interactions during bacterial infection.

## MATERIALS AND METHODS

### Bacterial strains and cell lines.

Fully virulent Y. pestis CO92, CO92 YopE-Bla, and CO92 Δ*pla* were obtained from the laboratory of William E. Goldman (University of North Carolina at Chapel Hill). *pgm*-negative strain CO92 YopE-Bla and CO92 Δ*pla* YopE-Bla were generated by bacterial conjugation as previously described (26). All experiments with these strains (with the exception of *pgm*-negative CO92 YopE-Bla) were performed under biosafety level 3 containment. Y. pestis strains were grown on brain heart infusion (BHI) agar (Difco) at 26°C for 2 to 3 days. For infection, Y. pestis CO92 and its derivatives were grown in 10 ml BHI broth containing 2.5 mM CaCl_2_ for 16 h at 37°C with constant shaking. THP-1 cells were obtained from ATCC and cultured in RPMI (Corning) containing 10% fetal bovine serum (FBS; Gibco) and antibiotic-antimycotic (Gibco). All cells were cultured at 37°C in 5% CO_2_.

### Preparation of human precision-cut lung slices and human alveolar macrophages.

Lungs obtained from anonymous donors through the Arkansas Organ Recovery Agency were perfused with phosphate-buffered saline (PBS), and the lobes were surgically separated. The major bronchi were cannulated, and the lobes were inflated with sterile 1.8% low-gelling-temperature agarose at 37°C. The bronchi were clamped and incubated at 4 to 7°C to allow the agarose to solidify. The hardened lungs were cut into 12-mm-thick sections with an 8.5-mm-diameter coring tool. Slices of ∼200 to 300 μm were prepared from these sections and cultured in 48-well plates in Dulbecco modified Eagle medium–Ham’s F-12 medium (DMEM-F12; 1:1) supplemented with 10% FBS, antibiotic-antimycotic, and an antibiotic formulation (Primocin) at 37°C in 5% CO_2_. For human alveolar macrophages (hAMs), PBS was used to lavage the lobes of donor lungs after cannulation. The lavage fluid was centrifuged at 500 × *g* for 10 min, treated with red blood cell lysis buffer (0.15 M NH_4_Cl, 12 mM NaHCO_3_, 0.1 mM EDTA) for 5 min, washed with PBS, and finally, reconstituted in DMEM-F12 (1:1) supplemented with 10% FBS and antibiotic-antimycotic. Cells were counted, plated in 12-well plates, and incubated at 37°C in 5% CO_2_ for 1 to 1.5 h to allow the hAMs to adhere. After incubation, fresh medium was added to the plates. hAMs and hPCLS were cultured for 4 to 5 days before they were used for infections.

### Infection of hPCLS and cells.

hPCLS were infected with 10^5^ or 10^7^ wild-type strain CO92 or CO92 Δ*pla* bacteria for 0.5 to 1 h in DMEM-F12 (1:1) supplemented with 10% FBS at 37°C in 5% CO_2_. After infection, the slices were washed 3 times with 1× PBS and flooded with medium before they were reincubated for the required time. To enumerate the CFU, hPCLS were digested in collagenase solution (1.5 mg/ml collagenase, 0.4 mg/ml DNase I, 10 mM HEPES, 5% FBS in Hanks balanced salt solution), serially diluted, and plated. To assess the viability of hPCLS, single-cell suspensions obtained from digested hPCLS were stained with CCF2-AM for 30 min at room temperature and processed for flow cytometry. hAMs and THP-1 cells were seeded at 10^5^ or 5 × 10^5^ cells/ml in 12-well plates. THP-1 cells were treated with phorbol 12-myristate 13-acetate (PMA; 100 ng/ml) for 24 h prior to infection. The cells were infected at a multiplicity of infection (MOI) of 20 for 1 h at 37°C in 5% CO_2_ for all assays. The cells were then washed 3 times with the respective medium and either reincubated or treated immediately according to assay requirements. To enumerate the number of CFU of surviving bacteria from hAMs, cells were infected at an MOI of 10 for 1 h, washed, and lysed with 0.1% Triton X-100 at the designated time points. The lysed cells were serially diluted and plated on BHI plates. The 0-h infection was plated immediately after adding bacteria.

### Cytokine measurements.

For hPCLS, infections were performed as described above. Culture supernatants were filtered through 0.22-μm-pore-size filters and stored at −80°C until use. All cytokines were measured using cytometric bead array kits (BD Biosciences) following the manufacturer’s instructions. The human inflammatory cytokines kit (IL-8, IL-1β, IL-10, IL-6, TNF-α, and IL-12p70) and human Th1/Th2/Th17 cytokine kit (IL-2, IL-4, IL-6, IL-10, TNF-α, gamma interferon, and IL-17A) were used.

### Flow cytometry.

hPCLS were digested in collagenase solution for 1 h at 37°C and gently teased apart with forceps before passing the suspension through a 70-μm-mesh-size cell strainer. Three hPCLS were typically pooled as a single sample for analysis that yielded roughly 10^5^ total cells, on average. The suspension was then centrifuged at 500 × *g* for 5 min, washed once, and resuspended in 1× PBS with 3% FBS (flow buffer) and fluorescent antibodies. The following antibodies were used at a 1:250 dilution: CD45-phycoerythrin (BD Biosciences), CD206-phycoerythrin-Cy7 (clone 19.2; eBioscience), CD71-Alexa Fluor 700 (clone M-A712; BD Biosciences), CD11c-Brilliant Violet 786 (clone B-ly6; BD Biosciences), CD16-Brilliant Violet 605 (clone 3G8; BD Biosciences), CD24-phycoerythrin CF594 (clone ML5; BD Biosciences), and CD14-allophycocyanin (clone M5E2; BD Biosciences). Cells were incubated at 4°C for 30 min, 1 ml of flow buffer was added, and the cells were centrifuged as described above. Cells were resuspended in 1× CCF2-AM (prepared per the manufacturer’s instructions; Invitrogen) with 50 μg/ml gentamicin and incubated at room temperature for 30 min. CCF2-AM also serves as a live/dead stain. Cells were washed once with flow buffer and fixed with 2% formalin in 1× PBS for 15 min at room temperature. Finally, cells were resuspended in flow buffer containing gentamicin and removed from the biosafety level 3 laboratory for flow cytometry. Cell populations were identified as follows (29): alveolar macrophages were CD45^+^ CD206^+^ CD71^+^, interstitial macrophages were CD45^+^ CD206^+^ CD71^−^, classical monocytes were CD45^+^ CD206^−^ CD71^−^ CD14^+^, dendritic cells were CD45^+^ CD206^−^ CD14^−^ CD11c^+^, and neutrophils were CD45^+^ CD206^−^ CD24^+^ CD16^+^. For BALF cells, the following antibodies were used at a 1:500 dilution: CD45-phycoerythrin (clone 30-F11; BD Biosciences), CD3-allophycocyanin-Cy7 (clone 17A2; BD Biosciences), CD11b-Alexa Fluor 700 (clone M1/70; BD Biosciences), CD11c-Brilliant Violet 786 (clone HL3; BD Biosciences), and F4/80-allophycocyanin (clone BM8; eBioscience). Alveolar macrophages were identified as CD45^+^ CD3^−^ F4/80^+^ CD11c^high^ CD11b^mid/low^ cells, interstitial macrophages were identified as CD45^+^ CD3^−^ F4/80^+^ CD11c^low^ CD11b^high^ cells, neutrophils were identified as CD45^+^ CD3^−^ F4/80^−^ Ly-6G^+^ CD11b^high^ cells, and dendritic cells were identified as CD45^+^ CD3^−^ F4/80^−^ CD11c^high^ CD11b^high/low^ cells (26). For flow cytometry of THP-1 cells and hAMs, infected cells were incubated with CCF2-AM for 30 min at room temperature, washed once, and resuspended in flow buffer.

### Microscopy.

For epifluorescence microscopy, hPCLS or hAMs were treated with CCF2-AM (prepared per the manufacturer’s instructions) for 30 min at room temperature, washed with PBS, and imaged under UV light in a Nikon Eclipse TS100 inverted microscope using a 4×, 10×, or 20× objective. For confocal microscopy, hPCLS were treated with 0.5% bovine serum albumin (BSA) and 0.3% Triton X-100 for 1 h at room temperature, followed by treatment with primary antibody (a 1:1,000 dilution of anti-human CD71; clone H68.4; Invitrogen) for 1 h. hPCLS were washed and treated with a 1:1,000 dilution of Alexa Fluor 488-conjugated secondary antibody (Alexa Fluor Plus 488; Invitrogen) for 1 h at room temperature. Finally, hPCLS were washed and mounted on glass-bottom petri dishes using ProLong Gold antifade reagent (Invitrogen). A Nikon Ti-Eclipse confocal microscope was used for imaging, and images were analyzed with NIS Elements software.

### Adherence assays.

Cells were infected as described above and processed as follows: after infection, one set of wells was washed with 1 ml 1× PBS 4 times and treated with 0.1% Triton X-100 for 5 min, followed by suspension in a final volume of 1 ml 1× PBS, while another set was scraped and suspended in 1 ml 1× PBS without washing. Both sets were serially diluted and plated on BHI plates to enumerate the CFU. Percent adherence was calculated as the number of bacteria in the washed wells divided by the total number of bacteria in the unwashed wells (25).

### Animal infections.

All animal experiments were conducted with approval from the UAMS institutional animal ethics committee. Female C57BL/6 mice, 6 to 8 weeks old, were obtained from The Jackson Laboratory and housed in cages with high-efficiency particulate air filters in biosafety cabinets. Animal housing temperatures were maintained at 25 to 26°C with 40% humidity. Bacteria were grown as described above and washed once with PBS. Mice were infected with 10^4^ CFU via intranasal inoculation with bacteria suspended in a 20-μl volume of 1× PBS. At the required time points, mice were sacrificed via intraperitoneal injection of sodium pentobarbital (150 mg/kg of body weight). Lungs were harvested, homogenized in 1 ml PBS, serially diluted, and plated on BHI agar for CFU enumeration. Bronchoalveolar lavage fluid (BALF) was obtained by tracheal cannulation as described previously (50). Briefly, euthanized mice were bled by cutting the hepatic portal vein. The trachea was cannulated using a 22-gauge catheter, and the lungs were inflated with 1 ml 1× PBS. The PBS was aspirated slowly, and the process was repeated 2 times. The BALF was centrifuged at 500 × *g* for 5 min, and the cells were treated with red blood cell lysis buffer for 1 min and diluted to 10 ml with PBS before centrifuging again. The cells were then processed for flow cytometry. For CFU enumeration from BALF, suspensions were serially diluted and plated on BHI agar.

### Statistical analyses.

All statistical analyses were done using the unpaired Welch’s *t* test. Results with *P* values of <0.05, <0.001, and <0.0001 are indicated in the figures. All statistical analyses were performed using GraphPad Prism (v7.04) software.
